# Life’s Essential 8, cardiovascular health, and incident dementia in diverse US adults of the Northern Manhattan Study

**DOI:** 10.1002/alz.088299

**Published:** 2025-01-09

**Authors:** Tali Elfassy, Hannah Gardener, Jose Gutierrez, Christian Agudelo, Clinton B. Wright, Mitchell S.V. Elkind, Bonnie Levin, Tatjana Rundek

**Affiliations:** ^1^ University of Miami Miller School of Medicine, Miami, FL USA; ^2^ Evelyn F. Mcknight Brain Institute, Miami, FL USA; ^3^ Evelyn F. McKnight Brain Institute, Miami, FL USA; ^4^ Vagelos College of Physicians and Surgeons, Columbia University, New York, NY USA; ^5^ NIH, Bethesda, MD USA

## Abstract

**Background:**

The American Heart Association’s Life Essential 8 (LE8) are a set of eight simple health metrics used to define cardiovascular (CV) health and promote healthy behaviors. High CV health is associated with a lower risk of CV disease. Our objective is to determine whether LE8 and CV health are associated with incident dementia in diverse adults.

**Methods:**

We used data from 877 participants of the Northern Manhattan Stroke study, a population based multi racial/ethnic cohort of adults age 40 and older. Information was collected on behavioral factors (diet, smoking status, physical activity, sleep duration) and clinical factors (BMI, blood pressure, cholesterol, fasting glucose, and medication use) which were measured and used to estimate a LE8 score (range: 0 to 100). CV health was defined as low (LE8 < 50), moderate (50<LE8≤80) or high (LE8≥ 80). Incident dementia was adjudicated during follow‐up by neuropsychological assessments. We used logistic regression analyses to assess the association between LE8 and CV health with incident dementia adjusting for age, sex, race/ethnicity, and education.

**Results:**

Mean age in the sample was 64 (SD: 8.2), 59.9% were female, 15.4% were non‐Hispanic White, 17.8% were non‐Hispanic Black, and 64.7% were Hispanic. Mean LE8 was 60.8 (SD: 12.4) and 18% had low CV health, 78.9% had moderate CV health, and 6.0% had high CV health. Over an average of 8.9 years of follow‐up, 145 people developed dementia. From multivariable adjusted models, each 10 unit decrement (worse LE8 score) was associated with a 19% higher odds of developing dementia (OR: 1.19, 95% CI: 1.02, 1.40). Compared with high CV health, odds of dementia was greater by 203% among those with low CV health (RR: 3.03, 95% CI: 1.07, 8.56), but not among people with moderate CV health (OR: 1.46, 95% CI: 0.55, 3.93).

**Conclusion:**

Only 1 in 20 adults in the study had high CV health. Poor CV health defined by the new LE8 metric substantially increases risk for developing dementia. Our results show that improvements in CV health factors are critical for prevention of dementia and further studies on health metrics are warranted.

